# Prognostic significance of a normal karyotype in adult patients with BCR-ABL1-positive acute lymphoblastic leukemia in the tyrosine kinase inhibitor era

**DOI:** 10.6061/clinics/2020/e2011

**Published:** 2020-11-02

**Authors:** Ting Shi, Huanping Wang, Mixue Xie, Xueying Li, Lixia Zhu, Xiujin Ye

**Affiliations:** I Department of Hematology, The First Affiliated Hospital of Medical School of Zhejiang University, Hangzhou, Zhejiang Province, China; II Key Laboratory of Hematology Oncology Diagnosis and Treatment of Zhejiang Province, Hangzhou, Zhejiang Province, China; III Program in Clinical Medicine, School of Medicine of Zhejiang University, Hangzhou, Zhejiang Province, China

**Keywords:** Acute Lymphoblastic Leukemia, BCR-ABL1, Philadelphia Chromosome, Normal Karyotype, Cytogenetics, ABL1 Mutation, Prognosis

## Abstract

**OBJECTIVE:**

The occurrence of cryptic Philadelphia (Ph) chromosome translocation is rare in BCR-ABL1-positive acute lymphoblastic leukemia (BCR-ABL1^+^ ALL) and is of unknown significance in the tyrosine kinase inhibitor (TKI) era.

**METHODS:**

We retrospectively studied a series of adult patients receiving TKI-based therapy to evaluate the prognostic impact of the normal karyotype (NK) (n=22) in BCR-ABL1^+^ ALL by comparison with the isolated Ph^+^ karyotype (n=54).

**RESULTS:**

There were no statistically significant differences in clinical characteristics and complete remission rate between the two groups. Compared with the isolated Ph^+^ group, the NK/BCR-ABL1^+^ group had a higher relapse rate (55.0% *versus* 29.4%, *p*=0.044). Overall survival (OS) and disease-free survival (DFS) were significantly shorter in the NK/BCR-ABL1^+^ group than in the isolated Ph^+^ group [median OS: 24.5 *versus* 48.6 (months), *p*=0.013; median DFS: 11.0 (months) *versus* undefined, *p*=0.008]. The five-year OS and DFS for patients with NK/BCR-ABL1^+^ were 19.2% and 14.5%, respectively; those for patients with isolated Ph^+^ were 49.5% and 55.7%, respectively. Thirty-four (44.7%) patients underwent allogeneic hematopoietic stem cell transplantation (allo-HSCT) in this study. Among the patients who received allo-HSCT, the median OS and DFS in the NK/BCR-ABL^+^ group (n=9) were 35.5 and 27.5 months, respectively, while those in the isolated Ph^+^ group (n=25) were undefined. There was a trend of significant statistical difference in the OS between the two subgroups (*p*=0.066), but no significant difference in the DFS. Multivariate analysis revealed that NK was independently associated with worse OS and DFS in BCR-ABL1^+^ ALL patients [Hazard ratio (HR) 2.256 (95% confidence interval (CI), 1.005-5.066), *p*=0.049; HR 2.711 (95% CI, 1.319-5.573), *p*=0.007].

**CONCLUSION:**

Our results suggest that the sub-classification of an NK could be applied in the prognostic assessments of BCR-ABL1^+^ ALL. In addition, allo-HSCT should be actively performed to improve prognosis in these patients.

## INTRODUCTION

Acute lymphoblastic leukemia (ALL) is a heterogeneous disease whose genetics are continuously evolving and will likely influence treatment decisions. Philadelphia (Ph) chromosome-positive, or BCR-ABL1-positive ALL, is a separate entity in ALL that accounts for 25-30% of adult cases and has an unfavorable prognosis (1,2). Cytogenetic analysis and molecular screening at the time of diagnosis showed that the results of these patients were quite heterogeneous, including some patients with the Ph chromosome as the sole abnormality, additional cytogenetic abnormalities, a complex-/variant-Ph chromosome, or a cryptic Ph chromosome with BCR-ABL1 rearrangement (Ph^-^/BCR-ABL1^+^). An ALL patient with Ph^-^/BCR-ABL1^+^ is quite rare and represents a unique clinical subtype. At present, the mechanism of cryptic Ph chromosome formation in BCR-ABL1^+^ leukemia is mainly derived from chronic myeloid leukemia (CML) research. Two major mechanisms were proposed: (1) there is a direct insertion between chromosome 9 and 22, that is, the proto-oncogene *ABL1* is directly inserted into the BCR region and *vice versa*, but the former is more common; and (2) there are two sequential translocations including the classic (9,22), followed by reverse translocation with each other, another chromosome, or both, thereby restoring the normal chromosome morphology (3,4).

At the time of disease diagnosis, cytogenetic analysis showed that the cryptic Ph chromosome translocation could present with a normal karyotype (NK) in approximately 3.0%-7.7% of CML patients, as well as a relatively small proportion of BCR-ABL1^+^ ALL patients (5-10). The prognosis of NK in CML patients has been reported, and the results in the tyrosine kinase inhibitor (TKI) era were controversial (4,). However, its role has not been systematically elucidated in adult patients with BCR-ABL1-positive ALL.

Therefore, we retrospectively analyzed the cytogenetic abnormalities in adult patients with BCR-ABL1^+^ ALL receiving chemotherapy plus TKIs to systematically investigate the different clinical implications for cases with NK/BCR-ABL1^+^ and isolated Ph^+^.

## MATERIALS AND METHODS

### Patients

We reviewed all patients who were newly diagnosed with BCR-ABL1^+^ ALL and had available data on conventional cytogenetics at diagnosis at our institution between 01/2010 and 12/2018. Only patients with an adequate number of analyzed metaphases (≥20) during conventional cytogenetic detection and receiving TKI-based therapy in the NK/BCR-ABL1^+^ (n=22) and isolated Ph^+^ (n=54) groups were included in the study (n=76). All the patients met the diagnostic criteria for ALL according to the World Health Organization classification. The patients underwent morphological examination, immunophenotype analysis by flow cytometry, cytogenetic analysis by routine R-banding with karyotype analysis, and leukemia fusion gene screening by multiplex nested reverse transcriptase-polymerase chain reaction (RT-PCR). Fluorescence *in situ* hybridization (FISH) was examined using BCR/ABL dual-color dual-fusion translocation Probes (Abbott, USA). Probe and slide preparation, as well as hybridization and washing steps, were performed according to the manufacturer’s protocols. Quantitative RT-PCR was used to determine the BCR-ABL1/ABL1 ratio, as well as the BCR-ABL1 positivity status during the whole process of treatment. Cytogenetic analysis for relapse or TKI resistance was performed at the discretion of the treating physician. The ABL1 kinase domain mutation analysis was conducted at the timing of relapsed or refractory status by direct Sanger sequencing. All patients were 16 years or older, and none of them had a history of malignant disease involving CML and myeloproliferative diseases. The study was approved by the Institutional Review Board of the First Affiliated Hospital of Zhejiang University. All procedures performed in studies involving human participants were in accordance with the ethical standards of the institution, national research committee, and 1964 Helsinki declaration and its later amendments or comparable ethical standards.

### Treatment and response definitions

All patients were treated with the standard VDCP±L,VDP±L, or Hyper-CVAD regimes plus TKIs, and chemotherapy comprising vincristine (V), daunorubicin or doxorubicin (D/A), L-asparaginase or asparaginase (L), cyclophosphamide (C), and steroid treatment (prednisone, or dexamethasone) (P/D). After complete remission (CR), intensive consolidation therapy was based on high-dose methotrexate, cytarabine, cyclophosphamide, and steroid treatment. The incorporation of TKIs into the therapeutic regime was continuous as maintenance therapy. Triple intrathecal therapy (methotrexate, cytarabine, and dexamethasone) was used for central nervous system prophylaxis. Allogeneic hematopoietic stem cell transplantation (allo-HSCT) was performed according to donor availability and the economic condition of the recipient. Response and relapse assessment were defined according to the ALL criteria of the National Comprehensive Cancer Network (NCCN) (14). Disease-free survival (DFS) was measured at the time of CR until relapse, death, or last follow-up (December 31, 2019). Overall survival (OS) was calculated from the time of disease diagnosis until death or last follow-up. Patients who were losing contact were censored at the last contact date. A total of 12 patients could not be contacted during the follow-up period. The median follow-up duration for the survivors was 21.0 months (range, 1.0-110.0 months).

### Statistical analyses

SPSS 23.0 software was used for the statistical analysis of these data. Descriptive statistics were used to illustrate the clinical features of these cases. Measurement data that conform to a normal distribution are expressed as the means±standard deviations, while non-normally distributed continuous data are expressed as the median (range). Categorical variables were compared by the chi-square test, and continuous variables were compared using the t-test or nonparametric analysis (Mann-Whitney U test). The Kaplan-Meier approach was used to estimate DFS and OS, and the log-rank test was used to compare survival estimates. A Cox regression model was used to identify prognostic variables in patients. Only variables with *p*<0.10 in the univariate analyses were included in the multivariate model; backward elimination was used until all variables showed a *p*-value of <0.05. All tests were double-tailed, and *p*<0.05 was considered statistically significant.

## RESULTS

This study included 40 (52.6%) men and 36 (47.4%) women, with an average age of 43.8 years (range, 16-76). The baseline characteristics of the two groups of patients are shown in Table 1. Cytogenetic heterogeneity was not correlated to patient gender, age, white blood cell count, hemoglobin, platelet count, ferritin level, lactate dehydrogenase level, or the type and ratio of BCR-ABL1/ABL expression. In terms of TKI options, three patients in the NK/BCR-ABL1^+^ and seven patients in the isolated Ph^+^ group were treated with second-generation TKIs (dasatinib) as the first-line treatment. In this study, 34 patients underwent allo-HSCT, and all achieved a post-remission state. Among patients receiving allo-HSCT, there were 9 patients (40.9%) in the NK/BCR-ABL^+^ group and 25 patients (46.3%) in the isolated Ph^+^ group. No statistically significant difference was observed in the treatment protocols and chemotherapy regimens between the two groups (*p*=0.668 and undefined, respectively).

**Figure 1:**
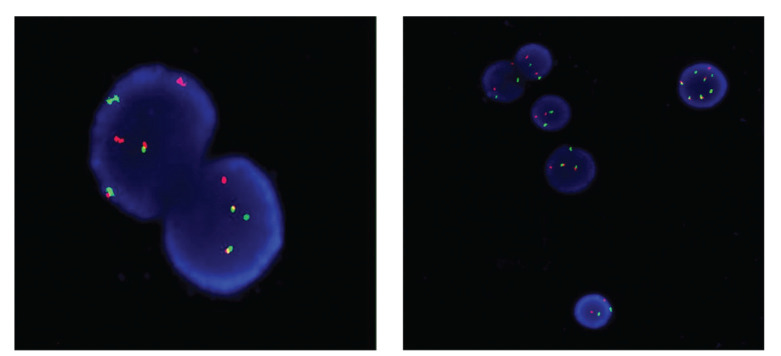
Fluorescence *in situ* hybridization (FISH) shows yellow fusion signal (BCR-ABL1 fusion gene) and separate red (ABL1), green (BCR) signals in interphase nuclei in two patients with normal karyotype (NK).

In the NK/BCR-ABL+ group, the bone marrow samples of two cases were used up and that of one case was of poor quality, resulting in the failure to obtain FISH results for these three patients. Of the remaining 19 patients, the typical abnormal signal pattern, involving chromosomes 22q11 and 9q34, were detected in 11 patients, and the complex pattern, which involves another chromosome in addition to 22q11 and 9q34, was detected in 8 patients (Fig. 1). There were only four patients in the NK/BCR-ABL1^+^ group who underwent cytogenetic examination at the time of relapse, and all presented with an NK. ABL1 kinase mutations were detected in 8 and 11 patients in the NK/BCR-ABL1^+^ and the isolated group, respectively. A spectrum of 9 types of amino acid substitutions at 8 different residues were detected, including T315I (n=12), E255K/V (n=3/3), G250E (n=1), Y253H (n=1), Q252H (n=1), E450A (n=1), F317L (n=1), and F486S (n=1). It is remarkable that T315I was, by far, the most frequent mutation, accounting for 63.2% (12/19) of all resistant cases. Of 19 patients with the ABL1 mutation, 14 (73.7%) patients had a single mutation, and 5 (26.3%) patients had two types of mutations. There was no statistical difference in the proportion of T315I mutation between the two groups. In the NK/BCR-ABL1^+^ group, the T315I mutation occurred in two patients after imatinib use, one patient after the first-line selection of dasatinib use, and three patients after the failure of imatinib treatment and subsequent switching to dasatinib use; in the isolated Ph^+^ group, the T315I mutation occurred in one patient after imatinib use, one patient after the first-line use of dasatinib, and four patients after the failure of imatinib treatment and subsequent switching to dasatinib or nilotinib use.

In this study, one patient in each of the two groups was losing c after the first induction chemotherapy, and one patient in the isolated Ph+ group died of intracranial hemorrhage during the first induction chemotherapy. The efficacy evaluation of the three patients was not completed. However, the efficacy in 73 patients, including 21 patients in the NK/BCR-ABL1^+^ group and 52 patients in the isolated Ph^+^ group, was evaluated. After the first induction therapy, 19 patients in the NK/BCR-ABL1^+^ group obtained CR (19/21, 90.5%), and 47 patients in the isolated Ph^+^ group obtained CR (47/52, 90.4%). All patients who selected dasatinib as the first-line treatment obtained CR after the first induction chemotherapy. There was no statistical difference in the CR rate between the two groups (*p*=1.000). Of 73 patients,1 patient in each group did not achieve remission after repeated induction chemotherapy, and follow-ups were conducted with 71 patients with regard to their recurrence. A total of 11 patients in the NK/BCR-ABL^+^ group had recurrences at a rate of 55.0%, and 15 patients in the isolated Ph^+^ group had recurrences at a rate of 29.4%, which was significantly lower than the corresponding values in the NK/BCR-ABL1^+^ group (*p*=0.044). In addition, there were 7 (7/11, 63.6%) and 8 (8/15, 53.3%) recurrent patients with the ABL1 mutation in the NK/BCR-ABL1^+^ and isolated Ph^+^ groups, respectively.

**Figure 2:**
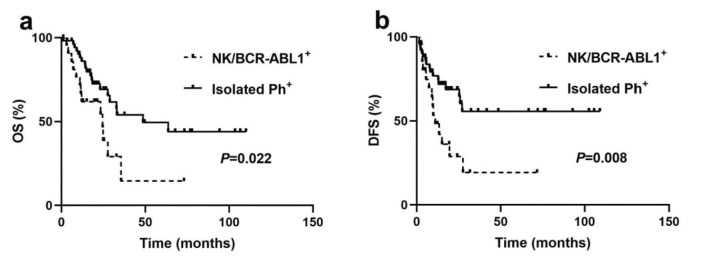
Outcomes for BCR-ABL1^+^ patients based on cytogenetic heterogeneity. (**a**) Overall survival (OS) for patients with NK/BCR-ABL1^+^ and isolated Ph^+^. (**b**) Disease-free survival (DFS) for patients with NK/BCR-ABL1^+^ and isolated Ph^+^.

The median OS and DFS of all patients were 32.8 months (range, 0.3-110.0 months) and 27.0 months (range, 1.5-109.0 months), respectively. The OS and DFS in the NK/BCR-ABL1^+^ group were significantly shorter than those in the isolated Ph^+^ group (median OS: 24.5 *versus* 48.6 months, *p*=0.022; median DFS: 11.0 months *versus* undefined, *p*=0.008) (Figure 2 a and b). The 5-year OS and DFS in the NK/BCR-ABL1^+^ group were 14.5% and 19.2%, respectively, while those in the isolated Ph^+^ subgroup were 49.5% and 55.7%, respectively.

**Figure 3:**
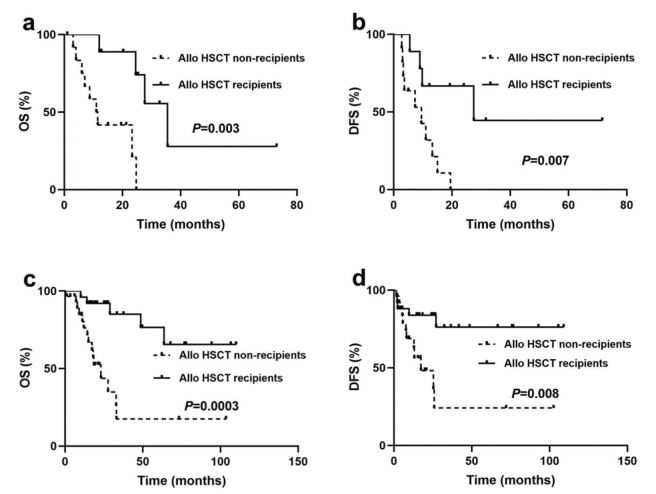
Outcomes for BCR-ABL1^+^ patients based on cytogenetic heterogeneity and stratified by treatment protocols. (**a**) Overall survival (OS) and (**b**) disease-free survival (DFS) for patients with NK/BCR-ABL1^+^ stratified by treatment protocols. (**c**) OS and DFS (**d**) for patients with isolated Ph^+^ stratified by treatment protocols.

The OS and DFS were longer in patients who underwent allo-HSCT than in those who did not (median OS: undefined *versus* 18.5 months, *p*<0.0001; median DFS: undefined *versus* 13.0 months, *p*=0.0002) (figure not shown). Further analysis of the survival difference between the two groups with or without allo-HSCT showed that the median OS and DFS of allo-HSCT patients in the NK/BCR-ABL1^+^ group were 35.5 and 27.5 months, respectively, and the median OS and DFS of patients who did not receive allo-HSCT were 11.3 and 9.5 months, respectively. The comparisons were statistically different (*p*=0.003 and *p*=0.007, respectively) (Figure 3 a and b).

Allo-HSCT patients in the isolated Ph^+^ group also had significantly better OS and DFS than patients who did not receive allo-HSCT (median OS: undefined *versus* 23.0 months, *p*=0.0003; median DFS: undefined *versus* 17.3 months, *p*=0.008) (Figure 3 c and d).

**Figure 4:**
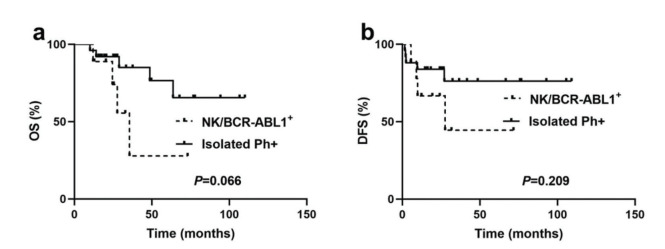
Outcomes for BCR-ABL1^+^ patients with receiving allo-HSCT based on cytogenetic heterogeneity. (**a**) Overall survival (OS) for patients with NK/BCR-ABL1^+^ and isolated Ph+. (**b**) Disease-free survival (DFS) for patients with NK/BCR-ABL1^+^ and isolated Ph^+^.

Of patients who underwent allo-HSCT, there was no significant difference in OS and DFS between the NK/BCR-ABL1^+^ group and the isolated Ph^+^ group (median OS: 35.5 months *versus* undefined; *p*=0.066; median DFS: 27.5 months *versus* undefined, *p*=0.209) (Figure 4 a and b).

Factors influencing OS and DFS in adult patients with BCR-ABL1^+^ ALL were analyzed by the univariate and multivariate cox regression analysis, as reported in Tables 2 and 3. As shown in Table 2, we found that the NK was the only independent risk factor for OS in adult patients with BCR-ABL1^+^ ALL (Hazard ratio (HR): 2.256; 95% confidence interval (CI): 1.005-5.066; *p*=0.049), while undergoing allo-HSCT was an independent protective factor for OS (HR: 0.196; 95% CI: 0.084-0.458; *p*<0.001). The results in Table 3 indicate that the NK was the only factor associated with worse DFS (HR: 2.711; 95% CI: 1.319-5.573; *p*=0.007), while undergoing allo-HSCT was the only factor associated with better DFS (HR: 0.224; 95% CI: 0.099-0.508; *p*<0.001).

## DISCUSSION

It is generally accepted that cytogenetic molecular abnormalities are well-recognized and powerful independent prognostic factors for numerous hematologic diseases. The Ph chromosome, creating a novel hybrid gene called BCR-ABL1, was associated with dismal outcomes in adult ALL patients in the pre-TKI era (2). In a few patients, the Ph chromosome translocation was cryptic, owing to some molecular-level insertions or translocations, and thus, could not be detected by G-banding chromosome analysis but could be identified by interphase-FISH and RT-PCR. This suggests that there may be genetic differences between the cryptic and the classic Ph chromosome, and its special formation mechanism may lead to heterogeneity in clinical characteristics and survival prognosis. At the time of disease diagnosis, the cytogenetic analysis showed that a proportion of Ph^-^/BCR-ABL1^+^ could be found in cases with an NK. Whether the clinical characteristics and prognosis of patients with Ph^-^/BCR-ABL1^+^ ALL or NK/BCR-ABL1^+^ ALL differ from those of Ph^+^ ALL is still unclear, and related reports are lacking.

In this study, we found that the NK/BCR-ABL1^+^ group was similar to the isolated Ph^+^ group, which corroborated the findings obtained in case of CML (12,15-17). With regard to the efficacy of TKIs and prognosis, there are only relevant studies for CML patients. Luatti et al. reported six cases of CML who presented with an NK during cytogenetic analysis at the time of diagnosis, and all the cases received imatinib therapy. Among them, four patients with low-risk Sokal scores achieved complete cytogenetic response at a median time of 24.5 months after receiving imatinib; one patient with a high-risk Sokal score developed imatinib resistance and demonstrated a major molecular response after switching to nilotinib; and only one patient with a moderate-risk Sokal score received allo-HSCT without remission and relapse (11). They concluded that the clinical benefit of TKIs in patients with NK/BCR-ABL1^+^ is similar to that in patients with Ph^+^, and this view is also recognized by Bennour et al. (13). A study published by Hochhaus et al. also found that like Ph^+^ patients, CML patients with an NK benefit from nilotinib, as these patients show similar molecular responses (12). On the contrary, three cases of CML with cryptic Ph chromosome reported by Haigh et al. failed to show a major cytogenetic response after imatinib therapy for at least three years, and two of them were shown to have an NK at the initial diagnosis via cytogenetic analysis (4). They believed that compared to classic Ph chromosomes, cryptic Ph chromosomes might confer a higher resistance to imatinib.

To our knowledge, this is the first study to examine the impact of an NK on prognosis in adult patients with BCR-ABL1^+^ ALL using a large mono-centric group of patients in the TKI era. Our analysis showed that despite a similar CR rate in patients with NK/BCR-ABL1^+^ and isolated Ph^+^, the former has a significantly higher recurrence rate. Our study also found inferior OS and DFS in patients with NK/BCR-ABL1^+^ compared to those with isolated Ph^+^. In addition, a trend of a worse prognosis for NK/BCR-ABL1^+^ patients was also observed in those receiving allo-HSCT. We also found that the NK was independently associated with a 2-fold higher risk of relapse or death in patients with BCR-ABL1^+^ ALL who are treated with chemotherapy plus a TKI. We analyzed the possible reasons for the worse outcome of NK/BCR-ABL1^+^ patients and found that the proportion of ABL1 kinase region mutations in the NK/BCR-ABL1^+^ group was 36.4%, of which T315I mutations accounted for 75%, which was slightly higher than the isolated Ph^+^ group (20.4% and 54.5%, respectively). In the first relapse patients, the proportion of patients in the NK/BCR-ABL1^+^ group with the ABL1 mutation was 63.6% (7/11), which is slightly higher than that in case of the isolated Ph^+^ group alone (53.3% (8/15)). The above results suggest that patients with NK/BCR-ABL1^+^ have cytogenetic instability, which may be an important cause of poor prognosis. We postulated that the higher rates of relapse seen in the NK/BCR-ABL1^+^ group might be, in part, due to an increased likelihood of TKI resistance in patients harboring an NK. Unfortunately, we cannot conclude the type and proportion of BCR-ABL1 mutation due to the small number of cases. Thus, future studies should integrate genomic profiling and ABL1 kinase mutation analysis to elucidate the mechanisms mediated by an NK that lead to inferior outcomes in patients with BCR-ABL1^+^ ALL. At the same time, it is worth exploring further whether there is a difference in the efficacy of each generation of TKIs.

Several limitations of the current study should be mentioned. Firstly, the sample size in the NK/BCR-ABL1^+^ group was relatively small. Secondly, the retrospective nature of this single-center analysis may inevitably affect the strength of the conclusion drawn. Multicenter retrospective analyses or prospective validation in a larger population size are warranted to further determine the role of the NK in BCR-ABL1^+^ ALL to guide complex management decisions.

Taken together, we demonstrated that the presence of an NK in patients with BCR-ABL1^+^ ALL receiving chemotherapy plus TKIs was associated with relatively poor outcomes and that an NK could be useful for risk stratification in the near future. Poor-risk NK should be taken into account when developing post-remission strategies in adult patients with BCR-ABL1^+^ ALL.

## AUTHOR CONTRIBUTIONS

Ting shi, Huanping Wang and Mixue Xie contributed equally to this work and share the first authorship. Conception and design: Ting Shi and Mixue Xie. Data collecting: Ting Shi. Statistical analysis: Lixia Zhu and Xueying Li. Manuscript writing: Ting Shi and Mixue Xie. Manuscript Revising: Ting Shi and Huanping Wang. Article reviewing: Xiujin Ye.

## Figures and Tables

**Table 1:** Patient characteristics.

Characteristics	All patients (n=76)	NK/BCR-ABL+ (n=22)	Isolated Ph^+^ (n=54)	*p*-value
Gender (n, M/F)	40/36	12/10	28/26	0.831
Age (ys, xḡ±s)	43.80±14.98	45.86±15.32	42.96±14.91	0.448
WBC count (*10E9/L, range)	14.10 (1.30-403.60)	13.15 (2.20-403.60)	16.20 (1.30-247.70)	0.208
Hb (g/L, xḡ±s)	102.74±26.42	105.11±19.58	101.78±28.86	0.622
PLT count (*10E9/L, range)	41.00 (2.00-405.00)	25.00 (8.00-309.00)	48.00 (2.00-405.00)	0.214
LDH (U/L, range)	513.00 (151.00-7209.00)	508.00 (164.00-4200.00)	513.00 (151.0-7209.00)	0.516
Ferritin (ng/ml, range)	794.45 (93.00-20985.50)	580.90 (93.00-3718.50)	862.80 (170.40-20985.50)	0.218
Blast in BM (%, range)	82.00 (22.00-96.00)	83.00 (37.50-96.00)	78.50 (22.00-96.00)	0.312
BCR-ABL/ABL (%, xḡ±s)	61.52±24.90	61.47±26.11	61.54±24.65	0.992
BCR-ABL isoform (n, p190/p210)	43/33	15/7	28/26	0.193
CNS involvement (n)	5	1	4	1.000
T315I mutation (n)	12	6	6	0.094
Treatment protocol (n)				
Chem+TKIs	42	13	29	0.668
Chem+TKIs+allo-HSCT	34	9	25	
Chemotherapy regimen (n)				
VDP±L	8	0	8	-
VDCP±L	61	19	42	
Hyper-CVAD	7	3	4	

Note: M—male; F—female; WBC—white blood cell; Hb—hemoglobin; PLT—Platelet; LDH—lactate dehydrogenase; BM—bone marrow; C—chemotherapy; CNS—central nervous system; TKIs—tyrosine kinase inhibitors; allo-HSCT—allogeneic hematopoietic stem cell transplantation.

**Table 2:** Risk Factors for Overall Survival in Univariate and Multivariate Analysis.

	Univariate Analysis			Multivariate Analysis		
Variable	HR	95% CI	*p*-value	HR	95% CI	*p*-value
Age [≥35/<35 (ys)]	1.856	0.834-4.131	0.130	-	-	-
WBC count [≥30/<30 (*10E9/L)]	1.510	0.739-3.081	0.258	-	-	-
PLT count [≥30/<30 (*10E9/L)]	0.851	0.427-1.696	0.647	-	-	-
Cytogenetics (NK/Isolated Ph+)	2.234	1.100-4.539	0.026	2.256	1.005-5.066	0.049
Disease relapse (yes/no)	3.036	1.476-6.245	0.003	1.895	0.815-4.406	0.138
CNS involvement (yes/no)	2.655	0.905-7.795	0.076	2.971	0.956-9.235	0.060
allo-HSCT (yes/no)	0.187	0.084-0.415	<0.001	0.196	0.084-0.458	<0.001

Note: WBC—white blood cell; PLT—platelet; CNS—central nervous system; allo-HSCT—allogeneic hematopoietic stem cell transplantation; CI—confidence interval; HR—Hazard ratio.

**Table 3:** Risk Factors for Disease-Free Survival in Univariate and Multivariate Analysis.

	Univariate Analysis			Multivariate Analysis		
Variable	HR	95% CI	*p*-value	HR	95% CI	*p*-value
Age [≥35/<35 (ys)]	1.844	0.788-4.314	0.158	-	-	-
WBC count [≥30/<30 (*10E9/L)]	1.563	0.745-3.278	0.237	-	-	-
PLT count [≥30/<30 (*10E9/L)]	1.083	0.528-2.221	0.827	-	-	-
Cytogenetics (NK/ Isolated Ph+)	2.524	1.239-5.145	0.011	2.711	1.319-5.573	0.007
CNS involvement (yes/no)	1.200	0.283-5.088	0.805	-	-	-
allo-HSCT (yes/no)	0.238	0.106-0.532	<0.001	0.224	0.099-0.508	<0.001

Note: WBC—white blood cell; PLT—Platelet; CNS—central nervous system; allo-HSCT—allogeneic hematopoietic stem cell transplantation.
